# Selenium Application During Radish (*Raphanus sativus*) Plant Development Alters Glucosinolate Metabolic Gene Expression and Results in the Production of 4-(methylseleno)but-3-enyl glucosinolate

**DOI:** 10.3390/plants8100427

**Published:** 2019-10-18

**Authors:** Marian McKenzie, Adam Matich, Donald Hunter, Azadeh Esfandiari, Stephen Trolove, Ronan Chen, Ross Lill

**Affiliations:** 1 The New Zealand Institute for Plant and Food Research, Ltd., Food Industry Science Centre, Private Bag 11600, Palmerston North 4442, New Zealand; adam.matich@plantandfood.co.nz (A.M.); Donald.hunter@plantandfood.co.nz (D.H.); Azadeh.esfandiari@plantandfood.co.nz (A.E.); Ronan.chen@plantandfood.co.nz (R.C.); Ross.lill@plantandfood.co.nz (R.L.); 2 The New Zealand Institute for Plant and Food Research, Ltd., Private Bag 1401, Havelock North 4157, New Zealand; Stephen.trolove@plantandfood.co.nz

**Keywords:** Selenium, Brassicaceae, glucosinolate, selenoglucosinolate, APS Kinase, selenate, indole, aliphatic

## Abstract

Selenium (Se) is an essential micronutrient for human health, entering the diet mainly through the consumption of plant material. Members of the Brassicaceae are Se-accumulators that can accumulate up to 1g Se kg^−1^ dry weight (DW) from the environment without apparent ill effect. The Brassicaceae also produce glucosinolates (GSLs), sulfur (S)-rich compounds that benefit human health. Radish (*Raphanus* *sativus*) has a unique GSL profile and is a Se-accumulating species that is part of the human diet as sprouts, greens and roots. In this report we describe the effects of Se-fertilisation on GSL production in radish during five stages of early development (from seed to mature salad greens) and on the transcript abundance of eight genes encoding enzymes involved in GSL metabolism. We tentatively identified (by tandem mass spectrometry) the selenium-containing glucosinolate, 4-(methylseleno)but-3-enyl glucosinolate, with the double bond geometry not resolved. Two related isothiocyanates were tentatively identified by Gas Chromatography-Mass Spectrometry as (*E/Z*?) isomers of 4-(methylseleno)but-3-enyl isothiocyanate. Se fertilisation of mature radish led to the presence of selenoglucosinolates in the seed. While GSL concentration generally reduced during radish development, GSL content was generally not affected by Se fertilisation, aside from the indole GSL, indol-3-ylmethyl glucosinolate, which increased on Se treatment, and the Se-GSLs, which significantly increased during development. The transcript abundance of genes involved in aliphatic GSL biosynthesis declined with Se treatment while that of genes involved in indole GSL biosynthesis tended to increase. APS kinase transcript abundance increased significantly in three of the four developmental stages following Se treatment. The remaining genes investigated were not significantly changed following Se treatment. We hypothesise that increased APS kinase expression in response to Se treatment is part of a general protection mechanism controlling the uptake of S and the production of S-containing compounds such as GSLs. The upregulation of genes encoding enzymes involved in indole GSL biosynthesis and a decrease in those involved in aliphatic GSL biosynthesis may be part of a similar mechanism protecting the plant’s GSL complement whilst limiting the amount of Se-GSLs produced.

## 1. Introduction

Selenium (Se) is an essential micronutrient for human health and a key element in the production of 25 selenoenzymes [1], such as glutathione peroxidase and selenoprotein P, which require Se at their catalytic site to be maximally active. Selenium largely enters the human diet through plants, which take up Se from the soil using the sulfur (S) uptake and assimilatory pathways [2]. While all plants take up Se, some plants accumulate Se in large quantities (up to 1g kg^−1^ DW) without apparent ill effects. These plants are known as Se-accumulators, or Se-indicators, and include many members of the Brassicaceae family, including radish [3]. Se-accumulators detoxify accumulated Se by the action of selenocysteine methyltransferase, which methylates selenocysteine (SeCys) to produce methylselenocysteine (MeSeCys) [4,5]. This reduces the non-specific incorporation of SeCys into proteins in place of cysteine (Cys), which can cause protein misfolding as Se atoms replace S [6]. In terms of human health, MeSeCys has been demonstrated to be beneficial, particularly in cancer prevention [7,8,9]. Therefore, there has been much interest in the production of Se-enriched Brassicaceae vegetables [3,10,11,12,13]. 

Brassicaceae are also well known for their ability to produce glucosinolates (GSL), S-rich secondary metabolites responsible for the pungent mustard flavour experienced when eating plants from this family [14,15]. The GSLs are a diverse group of compounds, estimated to date to be around 130 in number [16]. They are produced in a multistep process involving chain elongation of a base amino acid, biosynthesis of the core structure followed by side chain modification [17,18]. In discussions of GSL biosynthesis, it is relevant to group GSLs according to precursor amino acid. The best studied in the model plant Arabidopsis, and also those relevant in radish, are GSLs derived from Met and GSLs derived from tryptophan. The latter are also known as indole GSLs because they retain the indole group from tryptophan. The former are often referred to as aliphatic in discussions of biosynthesis, although it is not known whether all aliphatic GSLs follow the same pathways as those derived from methionine [18].

As well as being herbivory and pathogen deterrents [19,20], GSLs have clearly established health benefits for human health, particularly in cancer prevention [21]. GSLs themselves are biologically inactive, but they are converted to their biologically active aglycons [22,23] by the hydrolytic action of the β-thioglucosidase, myrosinase. Myrosinase is present in specialist plant cells [24] and is brought into contact with the GSLs during tissue breakdown, which occurs during herbivory or pathogen attack [19]. One group of aglycons, the isothiocyanates (ITCs), have been shown to induce mammalian Phase 2 enzymes and the apoptosis of cancer cells, and to interrupt the cell cycle, inhibiting cell proliferation [25]. 

The multiple health-related benefits of MeSeCys and the GSLs has led to increased interest in producing Brassicaceae with high amounts of both compound types [11,26]. However, this can be confounded by the chemical similarity of S and Se, resulting in substrates containing these elements effectively competing for the same enzymes. Se-fertilisation has been shown to reduce GSL production if Se is supplied in high concentrations [27,28]. Interestingly, when fertilised with Se, several Brassicaceae, including broccoli (*Brassica oleracea* L. var. *italica*), forage rape (*Brassica napus*) and cauliflower (*Brassica oleracea* L. var. *botrytis*), produce selenoglucosinolates (Se-GSLs), which have at least one S atom replaced by a Se atom [29,30,31]. These compounds are presumably produced by the incorporation of selenomethionine (SeMet) rather than Met as the base amino acid during GSL synthesis [29]. Elevating Se-GSLs in the accumulators may also be advantageous for human health, as there is evidence through in vitro studies that their selenated aglycons (synthetically produced) may have higher bioefficacy as a chemopreventative than their S counterparts [32]. 

Our interest is in understanding how and when Se-GSLs are produced during Brassicaceae development and in identifying Se-GSLs in tissues that have not been previously investigated, such as seed and sprouts. We determined the effect of Se-enrichment on GSL production in radish (*Raphanus sativus*), a species of Brassicaceae that is eaten at multiple developmental stages and which produces high concentrations of GSLs [33], including the aliphatic GSL 4MTB3 (4-(methylthio)but-3-enyl glucosinolate) that is found mainly in the *Raphanus* genus [34]. Appendix A shows the structures and names of GSLs commonly found in radish tissues. In addition to GSL content, we also investigated the transcript abundance of key genes involved in GSL biosynthesis and breakdown. We have tentatively identified a new Se-GSL, 4-(methylseleno)but-3-enyl glucosinolate (4MSeB3), and describe the altered expression of genes involved in aliphatic and indole GSL biosynthesis following Se fertilisation. We also suggest a mechanism that protects the plant’s GSL complement during Se fertilisation whilst limiting the amount of Se-GSLs produced. 

## 2. Results

### 2.1. Liquid Chromatography-Mass Spectrometry (LC-MS) Identification of a New Selenoglucosinolate

**Figure 1:**
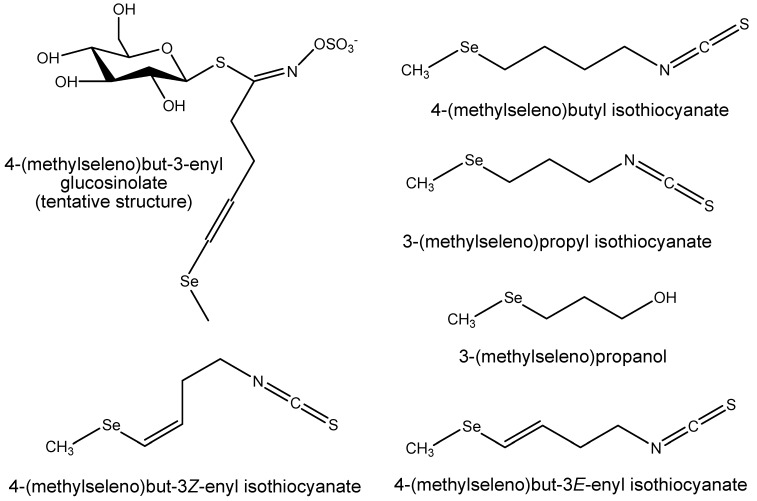
Selenium compounds tentatively identified in selenised *Raphanus sativus* seeds and sprouts.

**Figure 2:**
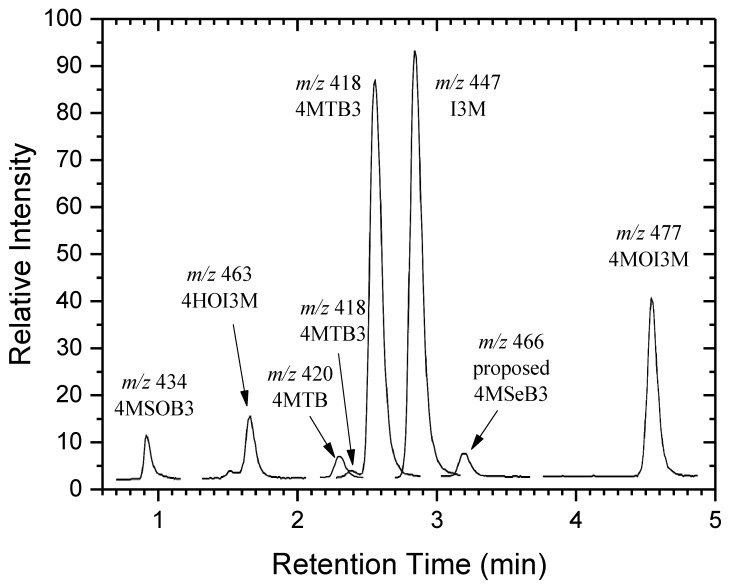
Reconstructed Liquid Chromatography-Mass Spectrometry (LC-MS) ion chromatogram of the pseudomolecular ions [M-H]^−^ of naturally occurring glucosinolates and putative seleno-glucosinolates identified in an ethanol extract of *Raphanus sativus* sprouts fertilised with selenium.

Figure 1 shows the structures of the selenium compounds we have tentatively identified in *Raphanus sativus* sprouts and seeds. In Appendix A we have included a table showing the structures of GSLs reported in radish tissues. In the present study we observed two GSL peaks, with pseudomolecular ions at *m/z* 418, in the chromatogram (2.43 and 2.6 min, Figure 2); we assigned these as geometric isomers of 4-(methylthio)but-3-enyl glucosinolate (4MTB3) [35]. The peak at 2.6 min had a pseudomolecular ion *m/z* 418.0304 ([M-H]^−^, −0.2 mDa, calcd. for C_12_H_20_NO_9_S_3_). 4MTB3 is a glucosinolate found mainly in the *Raphanus* genus and occurs as two geometric isomers, the major one being 4-(methylthio)but-(3*E*)-enyl glucosinolate [34,36]. We thus assumed the 2.6 min peak to be this (*E*)-isomer and the earlier eluting, minor peak at 2.43 min to be the (*Z*)-isomer (*m/z* 418.0292, [M-H]^−^, −1.4 mDa). 

The compound eluting at 3.25 min (Figure 2), 0.65 min after the presumed 4-(methylthio)but-3*E*-enyl glucosinolate, was tentatively identified as its selenised analogue 4-(methylseleno)but-3-enyl glucosinolate (4MSeB3). The presence of selenium in this compound was indicated by the complex selenium isotope pattern (Figure S1) of its pseudomolecular ion *m/z* 465.9749 ([M-H]^−^, +0.4 mDa, calcd. for C_12_H_20_NO_9_S_2_Se, 465.9745). 

**Figure 3:**
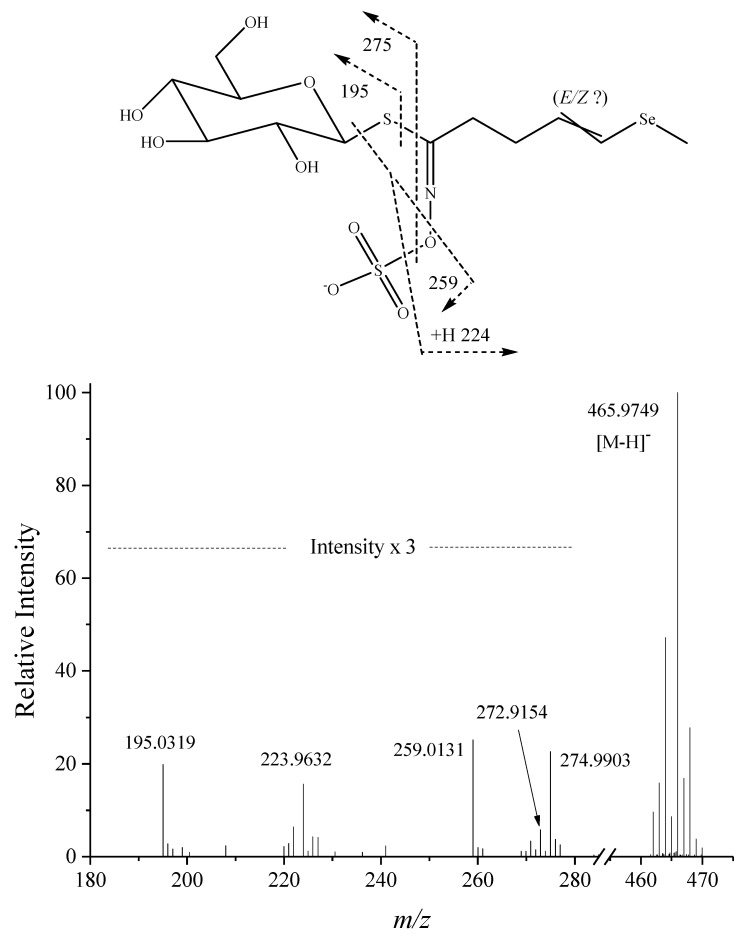
Fragmentation of the pseudomolecular ion ([M-H]^−^, *m/z* 466) of 4MSeB3 tentatively identified in an ethanol extract of *Raphanus sativus* sprouts fertilised with selenium. Ionisation energy was 26 eV and the collision cell gas was nitrogen. The daughter ions are shown at three-fold higher intensities, relative to the parent ion, than observed.

Fragmentation of this pseudomolecular (parent) ion was problematic (Figure 3) because ionisation energies capable of substantive fragmentation also fragmented the daughter ions. The low intensity of the daughter ions is shown in Figure 3 where they are displayed at three times their intensity relative to the parent ion. The data shown in this figure were obtained with a collision cell energy of 26 eV; at 27 eV, and higher, the parent ion was increasingly depleted with a concomitant decrease in the intensity of the daughter ions. At below 26 eV the intensity of the daughter ions suffered due to low fragmentation of the parent ion. This contrasts with the saturated compound 4MSeB for which we were able to produce quite intense daughter ions with respect to the parent ion [29].

There were two selenium-containing daughter ions; *m/z* 272.9154 ([C_6_H_9_O_3_S_2_Se]^−^, −0.9 mDa), which would correspond to the neutral loss of C_6_H_11_NO_6_ (mass 193). Assuming the glucose moiety to be intact, this neutral fragment might be nitroso-glucose. Such a fragment requires a complex rearrangement in order for nitrogen to attach to glucose. An alternate fragmentation might involve relocation of SO_3_^−^, onto the aglycon carbon atom adjacent to the bridging S atom to create a nitroso derivative, followed by elimination of an anhydroglucose molecule (C_6_H_10_O_5_) and a nitrosyl hydride molecule (HN=O). While there appeared a Se isotope cluster around this not very intense ion it was somewhat overshadowed by the nearby *m/z* 275 ion (Figure 3). The other Se-containing daughter ion was *m/z* 223.9632 ([C_6_H_10_NOSSe]^−^, −1.6 mDa), with a Se isotope cluster surrounding it Figure S1. This ion corresponded to rearrangement and neutral loss of C_6_H_10_O_8_S (mass 242), namely, the glucosyl-SO_3_ moiety deficient in one proton [37,38]. This ion is analogous to *m/z* 176 ([C_6_H_10_NOS_2_]^−^), a minor ion reported by Visentin et al. [34] for 4MTB3 by ammonia chemical ionisation, and to *m/z* 226 ([C_6_H_12_NOSSe]^−^) identified for the saturated analogue (4MSeB) of this compound [29]. 

In consideration of its selenisation and elution after the main isomer of 4MTB3 we concluded this compound could be 4-(methylseleno)but-3-enyl glucosinolate (4MSeB3). As the (*E*)-isomer of 4MTB3 dominates in the *Raphanus* genus, we propose this Se analogue is also the (*E*)-isomer. This identification relies on the supposition that in this Se compound the Se has replaced the S atom in the methylthio group. While there is biosynthetic reason for believing this to be the case, —namely, that the methylseleno moiety originates from SeMet—either isolation or synthesis of this compound for analysis by NMR is necessary to unambiguously prove this is so, and also to confirm placement and geometry of the double bond.

### 2.2. Gas Chromatography-Mass Spectrometry Identification of a New Methylselenoalkyl Isothiocyanate

**Figure 4:**
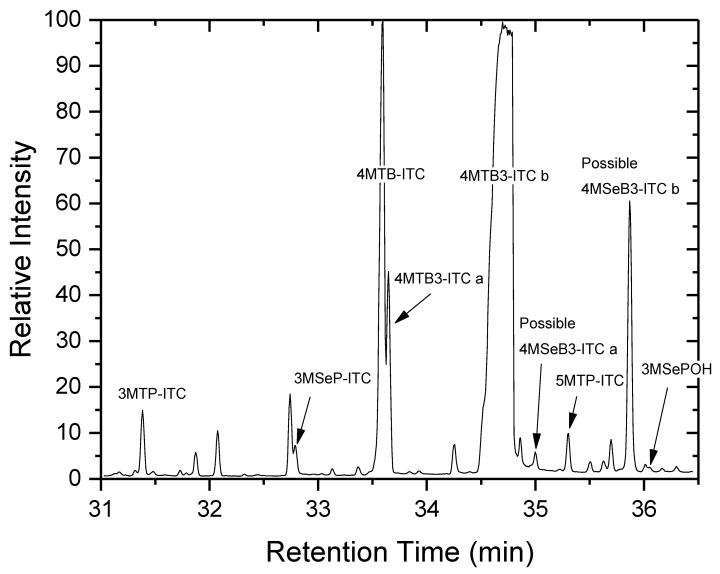
Total ion current Gas Chromatography-Mass Spectrometry (GC-MS) trace (on a DB-Wax capillary column) of isothiocyanate compounds identified in a diethyl ether extract of seeds harvested from *Raphanus sativus* plants fertilised with selenium.

In Figure 4 is a total ion chromatogram, of a diethyl ether extract of seeds harvested from *Raphanus sativus* plants fertilised with Se, showing the S isothiocyanates and their Se analogues. The major, heavily overloaded peak at ca. 34.7 min represents the compound identified, by its mass spectrum as 4MTB3-ITC and which we labelled as 4MTB3-ITC b (Figure S2, [39]). Eluting 1.05 min earlier at 33.65 min (and just after 4MTB-ITC) was a much smaller peak, 4MTB3-ITC a, with a very similar mass spectral fragmentation pattern (Figure S2). Based upon their relative abundances, the major latter eluting peak may be the (*E*)-isomer and the earlier peak the minor (*Z*)-isomer [36]. This elution order was reported by previous workers on a different polar column to that used herein [39].

**Figure 5:**
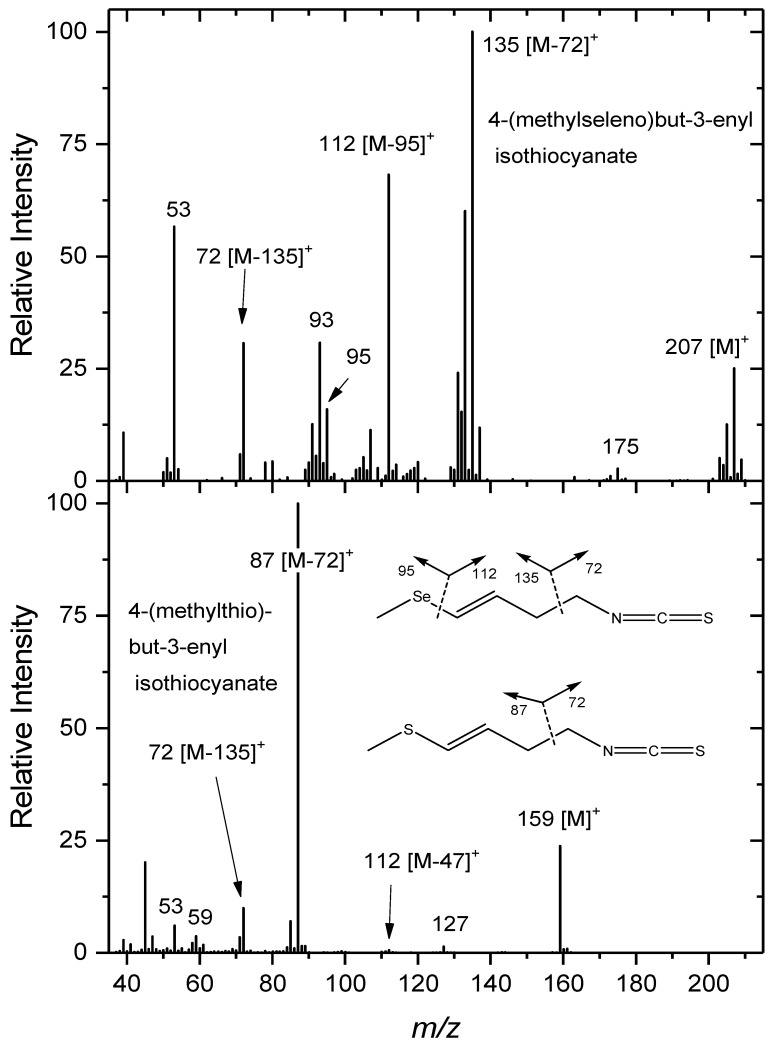
Comparison between the electron impact mass spectral fragmentation patterns of 4-MTB3-ITC and its proposed selenium analogue 4-MSeB3-ITC.

A pair of selenised compounds eluted after these two 4MTB3-ITC isomers; at 35.00 and 35.87 minutes (Figure 4). The first of these two selenised compounds eluted just after 4MSeB-ITC (not visible on the chromatogram) at 34.95 min. The two aforementioned selenised compounds (35.00 and 35.87 min.) have very similar mass spectra (Figure S3) which leads to the suggestion that they are isomers. In Figure 5 the EI fragmentation of the presumed Se isothiocyanate 4MSeB3-ITC b (RT = 35.87 min) is compared with that of the non-selenised compound 4MTB3-ITC. High resolution time-of-flight electron impact mass spectrometry (HR-ToF-EIMS) of the Se compound produced the molecular ion *m/z* 206.9639 ([C_6_H_9_NSSe]^+^, +1.8 mDa), *m/z* 134.9713 ([C_4_H_7_Se]^+^, 0.0 mDa), and *m/z* 92.9405 ([CHSe]^+^, +0.5 mDa). The ion, *m/z* 93 ([CHSe]^+^), has been observed previously for methylselenoalkyl compounds [29]. The base peak at *m/z* 135 (Figure 5) is suggestive of [CH_3_SeCH=CHCH]^+^, by analogy with the *m/z* 87 base peak of [CH_3_SCH=CHCH]^+^ from 4MTB3-ITC [39], and the presence of what appears to be an isotope cluster surrounding this peak.

In their identification of 4MTB3-ITC, Friis and Kjaer [39] reported the absence of a strong *m/z* 61, which corresponds to [CH_3_SCH_2_]^+^ in saturated methylthioalkyl isothiocyanates. This was viewed as support for the presence of a double bond adjacent to the methylthio group. Similarly, with the Se compounds herein, there exists no strong *m/z* 109 ([CH_3_SeCH_2_]^+^), but in saturated methylselenoalkyl isothiocyanates, identified previously, this ion was very strong [29]. Other fragment ions of note (but which we do not have accurate-mass data for) include *m/z* 72 ([CH_2_=NCS]^+^) which is a well-known isothiocyanate fragment [40], but which is reported to be either absent or present as a minor ion in the fragmentation pattern of alkylthiocyanates [41,42], and *m/z* 112 which may correspond to [CH=CHC_2_H_4_NCS]^+^. This fragment was a minor peak in the spectrum of the sulfur analogue, 4MTB3-ITC [39]. Similarly, *m/z* 114 ([C_4_H_8_NCS]^+^) was the base peak in the spectrum of the saturated compound 4MSeB-ITC [29], but in the S analogue, 4MTB-ITC, *m/z* 114 was a minor ion and the base peak was *m/z* 115 [40]. 

In summary, the above evidence suggests these selenised compounds are the (*Z*) and (*E*) isomers of 4-(methylseleno)but-3-enyl isothiocyanate. Fragmentation is consistent with the methylseleno- moiety adjacent to a double bond, and the other terminal functional group being an isothiocyanate. By analogy with the non-selenised compounds, the elution order on a polar GC column is the minor (*Z*)-isomer, followed by the major (*E*)-isomer. Confirmation of this assignment awaits isolation or synthesis for examination by NMR.

### 2.3. Selenium is Accumulated by the Seed of Radish Plants and by Radish Sprouts when Grown under Selenium Supplementation Conditions

Seed was harvested from radish plants that had been grown in sand either with Se supplementation (+Se seed) or without Se supplementation (−Se seed) and total Se content measured. Total Se content was low in −Se seed (0.1 µg g^−1^ DW) but was markedly higher (174 µg g^−1^ DW) in +Se seed (Table 1).

The total Se content in sprouts from +Se seed germinated in the absence of Se was similar to that in the +Se seed (Table 1). Germinating −Se seed in +Se water increased the Se content to greater than that of the +Se seed, and +Se seeds germinated in +Se water further increased the Se content of the sprouts to 335 µg g^−1^ DW (Table 1). 

### 2.4. Selenium Supplementation Enabled the Identification of the Selenoglucosinolate, 4-(methylseleno)but-3-enyl Glucosinolate, in Radish Seed and Sprouts

The glucosinolate profile of seeds from plants with and without Se enrichment and sprouts germinated from these seeds in the presence or absence of Se was determined by LC-MS analysis of ethanolic tissue extracts (Table 2). The major GSL detected in the seed was 4-(methylsulfinyl)but-3-enyl glucosinolate (4MSOB3), being approximately 10-fold greater than the combined isomers of 4MTB3, with 4-(methylthio)butyl glucosinolate (4MTB) the least prevalent. 

The GSL profile of 5-day-old sprouts was the same regardless of whether they were grown from +Se or –Se seed, or germinated in –Se or +Se water (Table 2). However, the concentration of 4MSOB3 was reduced in sprouts compared with seed and the converse was true for 4MTB3. 

In addition to the GSLs previously described for radish, we identified the selenoglucosinolate 4-(methylseleno)but-3-enyl glucosiniolate (4MSeB3) in seed and sprouts exposed to Se (Section 2.1, Table 2).

### 2.5. Selenised GSL Hydrolysis Products are also Found in Radish Seed and Sprouts

For both seeds and 5-day-old sprouts, the major GSL hydrolysis product was 4MTB3-ITC b, followed by 4MTB-ITC, 4MTB3-ITC a, 3-(methylthio)propyl isothiocyanate (3MTP-ITC) and 5-(methylthio)pentyl isothiocyanate (5MTP-ITC), although the latter was only detected in the sprouts (Table 3). We next considered GSL biosynthesis during radish plant development. 

### 2.6. Glucosinolate Content Reduces during Radish Development and is Generally not Affected by Selenium Treatment, except for indol-3-ylmethyl Glucosinolate and 4-methoxyindol-3-ylmethyl Glucosinolate

**Figure 6:**
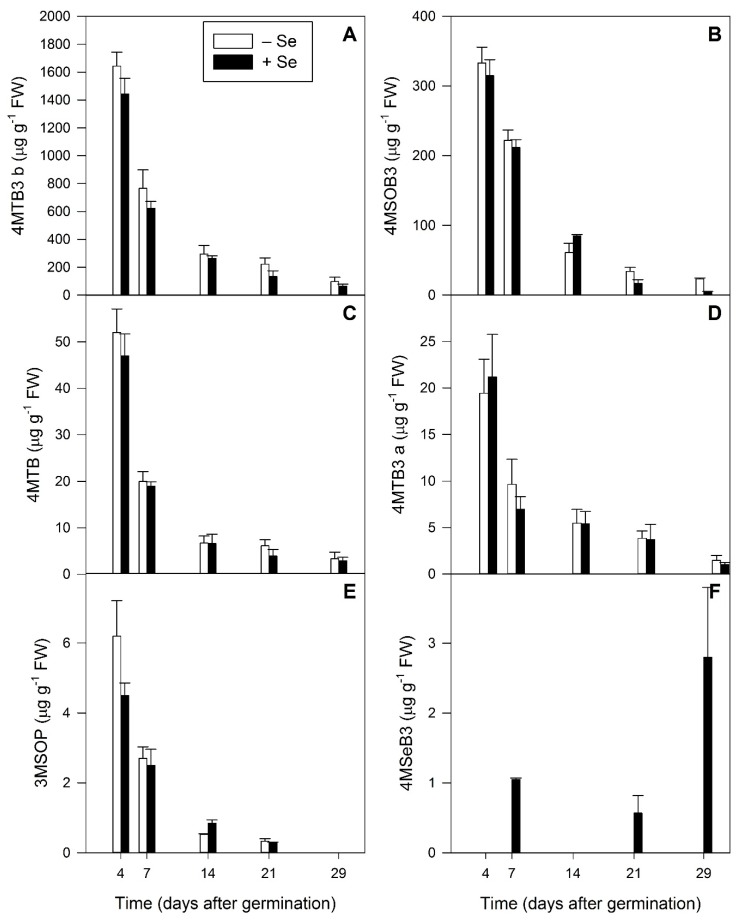
Aliphatic glucosinolates detected using LC-MS analysis of five developmental stages (4, 7, 14, 21 and 29 days after germination) of *Raphanus sativus* plants grown hydroponically in the absence (white bars) and presence (black bars) of 40 µM sodium selenate. Each bar represents the mean value of three biological replicates, n = 3. Error bars represent the standard error of the mean. (**A**) 4MTB3 b: 4-(methylthio)but-3-enyl isomer b, (**B**) 4MSOB3: 4-(methylsulfinyl)but-3-enyl, (**C**) 4MTB: 4-(methylthio)butyl, (**D**) 4MTB3 a: 4-(methylthio)but-3-enyl isomer a, (**E**) 3MSOP: 3-(methylsulfinyl)propyl, (**F**) 4MSeB3: 4-(methylseleno)but-3-enyl.

Radish plants were grown hydroponically, with or without selenate, and harvested at intervals corresponding to commercial sprout and greens products. The oldest plants, representing mature greens, were harvested 29 days after germination (DAG). LC-MS analysis confirmed the presence of all the aliphatic GSLs detected in the seeds and sprouts from the previous experiment. However, here 4MTB3 b was the predominant GSL, followed by 4MSOB3 and 4MTB (Figure 6, A–D). We also detected 3MSOP in low amounts (Figure 6E). In all cases the concentration of the aliphatic GSLs significantly decreased during development (*p* < 0.001, Figure 6, A–E). 

As with the previous experiment, the Se-containing GSL 4MSeB3 was detected at low concentrations in plants fertilised with Se (Figure 6F), starting from 7 DAG (sprouts with fully expanded cotyledons). The content of this Se-GSL, whilst remaining low, increased significantly during development to a maximum at 29 DAG (mature greens) (*p* = 0.022), which was the last developmental stage measured.

**Figure 7:**
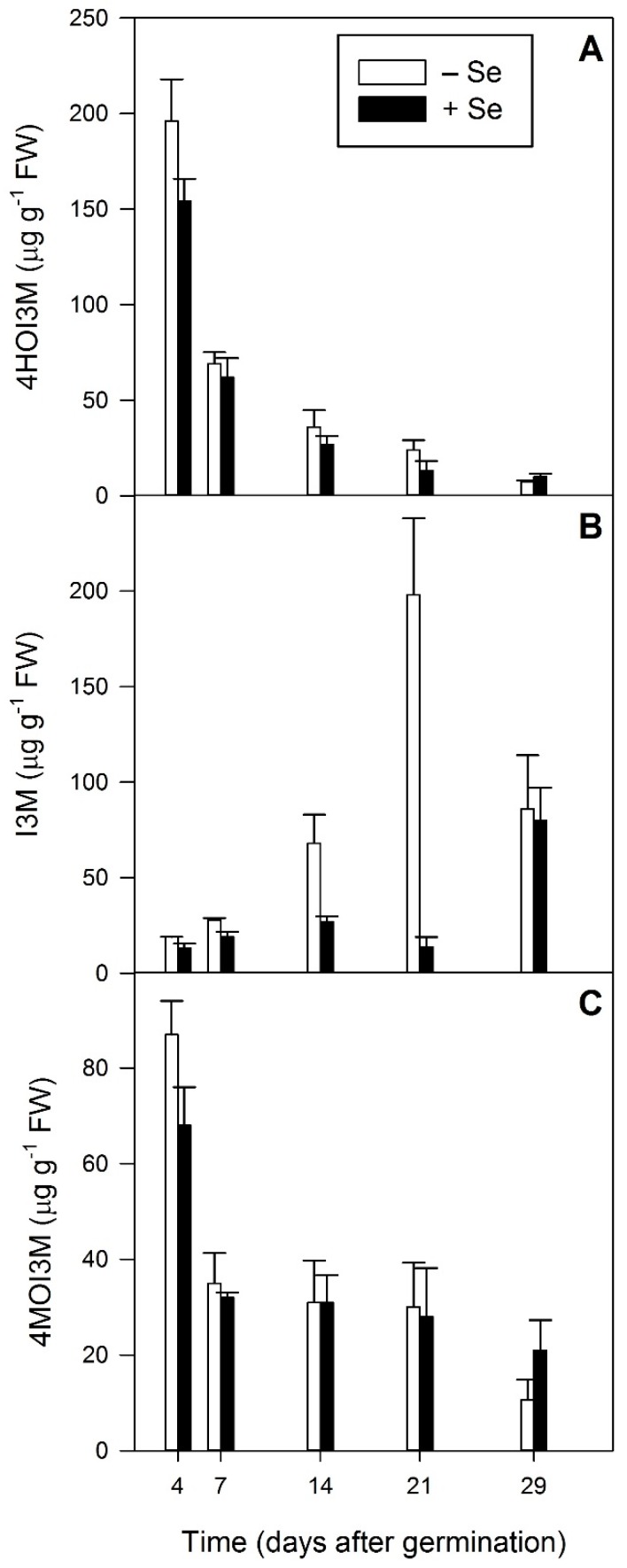
Indole glucosinolates detected using LC-MS analysis of five developmental stages (4, 7, 14, 21 and 29 days after germination) of *Raphanus sativus* plants grown hydroponically in the absence (white bars) and presence (black bars) of 40 µM sodium selenate. Each bar represents the mean value of three biological replicates, n = 3. Error bars represent the standard error of the mean. (**A**) 4HOI3M: 4-hydroxyindol-3-ylmethyl glucosinolate, (**B**) I3M: indol-3-ylmethyl glucosinolate, 4MOI3M: (**C**) 4-methoxyindol-3-ylmethyl glucosinolate.

Indole GSLs were also detected in the developing radish tissue, the predominant one being 4-hydroxyindol-3-ylmethyl glucosinolate (4HOI3M), followed by indol-3-ylmethyl glucosinolate (I3M) and 4-methoxyindol-3-ylmethyl glucosinolate (4MOI3M) (Figure 7). As observed for the aliphatic GSLs, 4HOI3M concentration steadily decreased with developmental stage (*p* < 0.001), and was not significantly affected by Se treatment (Figure 7A). In contrast, I3M significantly increased with developmental stage (*p* < 0.001, Figure 7B). This was particularly so for control plants at 14 and 21 DAG (microgreens and mature greens respectively). The I3M concentration in Se-treated plants also increased during development. However, at 14 and 21 DAG the concentration was significantly lower (*p* < 0.001) in Se fertilised plants compared to controls, though the contents were similar again by 29 DAG (Figure 7B). Of the GSLs measured, I3M was the only one to display this developmental pattern. Like the other GSLs, 4MOI3M declined during plant development, but its concentration tended to plateau at 7 DAG (fully expanded cotyledons) and there was no significant effect seen due to Se treatment (Figure 7C).

### 2.7. Glucosinolate Hydrolysis Products also Decrease during Radish Plant Development, except for the Selenised Forms

**Figure 8:**
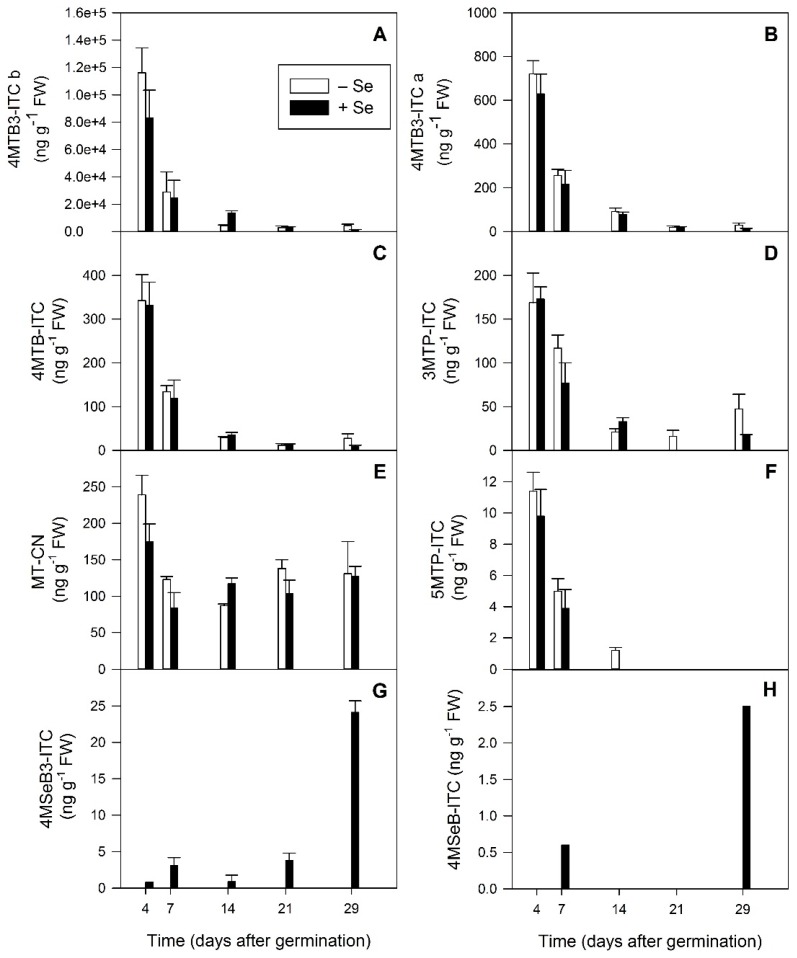
Isothiocyanate and nitrile glucosinolate breakdown products detected using GC-MS analysis of five developmental stages (4, 7, 14, 21 and 29 days after germination) of *Raphanus sativus* plants grown hydroponically in the absence (white bars) and presence (black bars) of 40 µM sodium selenate. Each bar represents the mean value of three biological replicates, n = 3. Error bars represent the standard error of the mean. (**A**) 4MTB3-ITC b: 4-(methylthio)but-3-enyl isothiocyanate isomer b, (**B**) 4MTB3-ITC a: 4-(methylthio)but-3-enyl isothiocyanate isomer a, (**C**) 4MTB-ITC: 4-(methylthio)butyl isothiocyanate, (**D**) 3MTP-ITC: 3-(methylthio)propyl isothiocyanate, (**E**) MT-CN: methylthiocyanide, (**F**) 5MTP-ITC: 5-(methylthio)pentyl isothiocyanate, (**G**) 4MSeB3-ITC: 4-(methylseleno)but-3-enyl isothiocyanate, (**H**) 4MSeB3-ITC: 4-(methylseleno)but-3-enyl isothiocyanate.

The concentration of all GSL hydrolysis products (Figure 8) decreased during radish development, except for methylthiocyanide (MT-CN, Figure 8E), which after an initial decline between 4 and 7 DAG, remained at a constant concentration for the rest of the experiment. Selenium treatment resulted in significantly higher concentrations of the major 4MTB3-ITC isomer at 14 DAG (microgreens) and significantly lower concentrations at 29 DAG (mature greens) (*p* < 0.001, Figure 8A). This was not observed for the minor 4MTB3-ITC isomer (Figure 8B). Significantly higher concentrations of 3MTP-ITC were seen in control plants at 21 and 29 DAG compared with Se-treated plants (*p* < 0.001, Figure 8D) a trend also observed for 5MTP-ITC concentrations at 14 DAG (*p* < 0.001, Figure 8F). 

Selenium treatment resulted in the production of 4MSeB3-ITC (Figure 8G) and 4MSeB-ITC (Figure 8H) at low concentrations. These compounds were also detected in sprouts from the previous experiment (Table 3). Like 4MSeB3, their parent compound, the concentrations of both these seleno-GSL hydrolysis products increased significantly during radish development to a maximum at 29 DAG (mature greens) (*p* = 0.002 and *p* = 0.05 respectively, Figure 8G,H). We would expect their concentration to increase further during development as biosynthesis of the selenoglucosinolates continued.

### 2.8. Selenium Treatment Increases the Transcript Abundance of Genes Involved in Indole GSL Production while Decreasing the Transcript Abundance of Genes Involved in Aliphatic GSL Production. 

**Figure 9:**
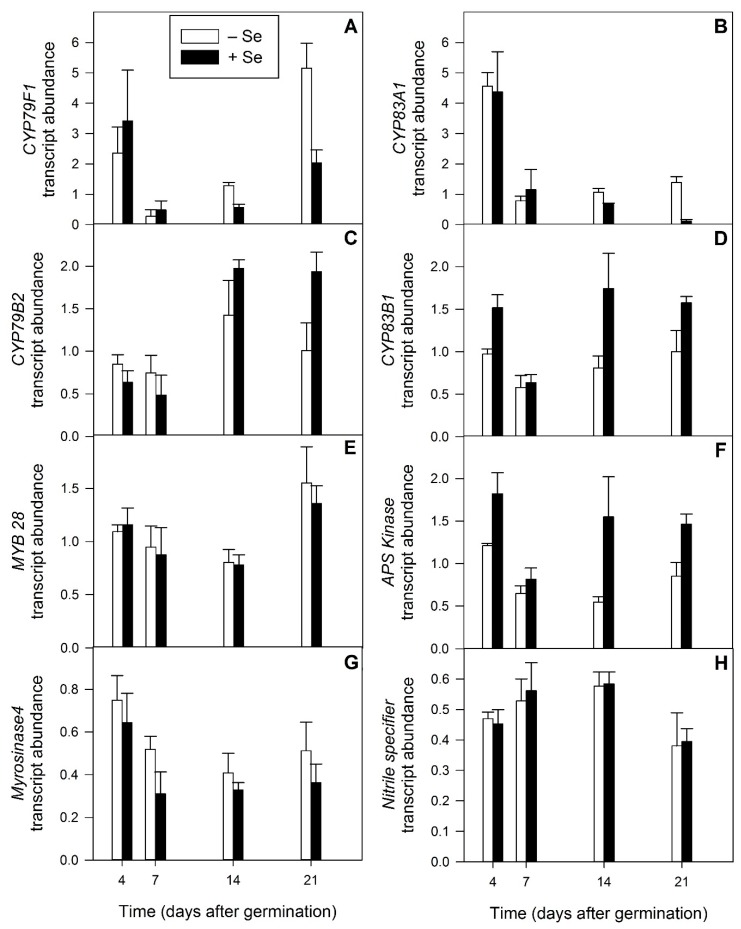
Transcript abundance of glycosinolate metabolism genes in *Raphanus sativus* plants at four developmental stages (4, 7, 14, 21 and 29 days after germination) grown hydroponically in the absence (white bars) and presence (black bars) of 40 µM sodium selenate. (**A**) *CYP79F1*, (**B**) *CYP83A1,* (**C**) *CYP79B2*, (**D**) *CYP83B1*, (**E**) *MYB28*, (**F**) *APS Kinase* (**G**) *MYROSINASE4*, (**H**) *NITRILE SPECIFIER*. Transcript abundance was normalised to *ACTIN7*. Each bar represents the mean value of three biological replicates, n = 3. Error bars represent the standard error of the mean.

To further investigate the timing of GSL biosynthesis in radish development and the effect of Se-fertilisation on the expression of several key GSL biosynthetic and metabolism genes, the transcript abundance of eight genes was investigated in the sprout and shoot tissue from radish plants from 4 to 21 DAG (sprout to salad greens) in the presence or absence of Se fertilisation (Figure 9). 

During radish development, a significant decrease in expression levels of *CYP79F1*, which encodes the first enzyme in the biosynthesis of the aliphatic core GSL structure, was observed from 4 to 14 DAG (young sprouts to microgreens, respectively, *p* = 0.001, Figure 9A). *CYP79F1* transcript abundance increased again after a further 7 days to a maximum at 21 DAG (salad greens) in the controls. This increase was also observed in Se-fertilised plants, but it was reduced compared with the controls. Transcript abundance of *CYP83A1*, encoding the second gene in the biosynthesis of the core aliphatic GSL structure, was also observed to significantly decline from 4 DAG and remained low for the rest of the experiment (*p* < 0.001, Figure 9B). In general, Se-fertilised plants had significantly lower *CYP83A1* transcript abundance than the controls (*p* = 0.001, Figure 9B). 

*CYP79B2* and *CYP83B1* encode key enzymes in indole GSL biosynthesis [18]. In our studies, *CYP79B2* gene expression increased significantly (*p* < 0.001) between 7 and 14 DAG (microgreens) and was maintained at 21 DAG (salad greens) (Figure 9C). Selenium fertilisation tended to further increase transcript abundance of this gene at both developmental time points, with the ‘Se treatment x DAG’ interaction term suggesting that this increase was significant (*p* = 0.026) by 21 DAG. The transcript abundance of *CYP83B1* remained similar throughout radish development for control plants. However, Se fertilisation significantly increased transcript abundance of this gene at 4, 14 and 21 DAG (*p* = 0.002, Figure 9D).

No difference was seen in the transcript abundance of *MYB28*, a major regulator of short-chain aliphatic GSL biosynthesis, except for a slight increase (*p* = 0.059) at 21 DAG for both control and Se-fertilised plants (Figure 9E). No change in expression of this gene was seen in response to Se fertilisation at any time point. 

In contrast, the transcript abundance of *APS Kinase* was highly significantly increased by Se fertilisation at three of the four developmental stages: sprouts, microgreens and salad greens (*p* < 0.001, Figure 9F). 

The transcript abundance of genes encoding myrosinase and nitrile specifier protein (NSP), both of which are involved in GSL breakdown and hydrolysis, was not significantly altered by Se fertilisation at any time during the developmental series (Figure 9G,H). These genes responded in an opposite manner during development, with *MYROSINASE4* transcript abundance decreasing to its lowest level at 14 DAG (microgreens), while *NSP* transcript abundance increased slightly over this same time period. These trends were not significant however.

## 3. Discussion

As expected, the total Se content of seeds from plants grown without Se supplementation (−Se seed) was low, but Se supplementation of the parent plant resulted in seed with increased Se content (+Se seed, Table 1). This is consistent with other studies that have shown that selenium accumulates in the seed of Se-fertilised plants [43,44,45], including those of the Brassicaceae [46,47,48,49]. However, sprouts with the highest Se content were achieved when seeds were germinated in the presence +Se water (Table 1). It would appear from these results that while pre-loading radish seed with Se is a valid method for fortifying radish sprouts with Se, increased Se content is most easily achieved by absorbing Se from the liquid the seed are germinated in. Although this method results in greater variability in the Se content of the sprouts, and Se treatment of the +Se seed during germination resulted in the highest Se content (Table 1). The Se enrichment method used should be based on the desired product outcome. For example, seed meal from Brassicaceae grown on Se-rich soil showed potential as a natural fertiliser to increase the Se content of organic strawberry fruit [46] and a single foliar spray at flowering time was a simple method to produce Se-enriched buckwheat and pumpkin seed [44]. 

LC-MS analysis of the GSL profile in the seed confirmed the major GSL was 4MSOB3, which was approximately 10-fold greater than the combined isomers of 4MTB3, and 4MTB was the least prevalent (Table 2). These glucosinolates have previously been described in radish seed [12,50], which are regarded as a major source of 4MSOB3 [51]. 

In 5-day-old sprouts germinated from this seed, the GSL content was the same regardless of what seed type they were grown from, or whether they were germinated in −Se or +Se water (Table 2). However, there was a shift in the relative proportions of 4MSOB3 and 4MTB3, which has previously been documented [52,53], as 4MSOB3 is chemically reduced to 4MTB3. 

The identification of the selenoglucosinolate 4MSeB3 in seed and sprouts exposed to Se (Section 2.1, Table 2), for the first time, is interesting. We have previously identified the Se-GSLs 3-(methylseleno)propyl glucosinolate, 4-(methylseleno)butyl glucosinolate, 5-(methylseleno)pentyl glucosinolate, 4-(methylseleninyl)butyl glucosinolate and seleno-2-phenylethyl glucosinolate in other members of the Brassicaceae family; broccoli, cauliflower and forage rape [29,30]. In the latter compound the location of the Se atom was not determined. This is the first identification of 4MSeB3, whose sulfur analogue, 4MTB3, is found in members of the *Raphanus* genus [35]. 4MSeB3 has a single Se atom in the side chain, replacing the S atom (Figure 1), indicating it is derived from SeMet in place of Met [18,54], and was detected only as a single geometric isomer. The (*E*)-isomer of the sulfur analogue, 4MTB3 b, is the major form at 40 to 100-fold more than the (*Z*)-isomer, 4MTB3 a, in the sprouts (Table 2). 

Glucosinolates are not synthesised by seed [55], instead being transported from maternal tissues by high-affinity GSL-specific transporters (GTR1 and GTR2) during seed development [56]. The current study suggests that these transporters also accept and transport the Se-GSLs.

In the sprouts, 4MSeB3 was only found in tissue germinated from +Se seeds, regardless of the presence of Se in the sprout germination water (Table 2). This suggests incorporation of Se into the GSLs does not occur during the first five days of sprout development, which is consistent with reports that there is no significant net GSL biosynthesis during the first days of germination [57,58,59,60]. However, when looked at on a dry weight basis our data indicates a substantial increase in total GSL during the 5-day germination period. All our data are presented on a fresh weight (FW) basis, because our focus was on the dietary portion. However, we should consider the dilution effect of the water uptake by the seeds. Borș et al. [61] report that DM% for radish seeds drops from 95% to 23.5% by 3 DAG, falling further to 13.7% by 5 DAG. The DM% of the 5-day-old sprouts in our experiment averaged 13.8%, therefore on a dry weight basis, the quantities of GSLs and their breakdown products present at 5 DAG (Table 2 and Table 3) were approximately 5-fold greater than those present in the seed.

In the seed, the concentration of hydrolysis products was low (Table 3), which contrasted markedly with the comparatively high GSL levels in the seeds (Table 2) and is suggestive of very little myrosinase activity therein. It is also worth noting that the seed sourced from selenised radish plants had considerably more (up to 37-fold for 4MTB-ITC) of the non-selenised GSL products than seeds sourced from non-selenised radish plants. This reflects the trend seen for increased GSL content in the +Se seed (Table 2); although this observation could not be verified for significance by statistical analysis as only one replicate of 2–3g seed was analysed in each case. In sprouts, the quantities of the hydrolysis products were similar regardless of the seed source or whether the seeds were germinated in the presence of absence of Se (Table 3). The aglycon 4MTB3-ITC is a hydrolysis product of 4MTB3, and 4MTB-ITC is a hydrolysis product of 4MTB.

It was interesting that in this study we did not observe 4-(methylsulfinyl)but-3-enyl isothiocyanate (4MSOB3-ITC), a hydrolysis product of 4MSOB3, nor its Se analogue, 4MSeOB3-ITC. Given the high concentration of 4MSOB3 measured in the tissues, observation of the non-selenised 4MSOB3-ITC would be expected. It is probable that if present in the samples, under the chromatographic conditions used, these compounds eluted after the 45 min run time used on the polar GC column employed. Despite the above caution, it is probable that the selenised compound 4MSeOB3-ITC was not present, because we did not identify its parent seleno-GSL, 4MSeOB3. In a previous study we attempted to discover the chemically related compound, 4-(methylseleninyl)butyl glucosinolate (4MSeOB), in high GSL broccoli and were only able to find it as a trace amount of less than 0.1% of its S analogue, 4-(methylsulfinyl)butyl glucosinolate. It was concluded that the selenoxide moiety is not chemically stable [30].

The detection of selenised GSL hydrolysis products in sprouts germinated from +Se seed (Table 3) was not surprising considering the identification of Se-GSLs in this tissue (Table 2). The major selenised hydrolysis product was 4-(methylseleno)but-3-enyl isothiocyanate (4MSeB3-ITC), reflecting the prevalence of its non-selenised form. However, it is of interest that 3-(methylseleno)propyl isothiocyanate (3MSeP-ITC) was detected but not its parent GSL, 3-(methylseleno)propyl glucosinolate, and not all of the selenised hydrolysis products were versions of the non-selenised forms. For example, in the case of 3MSePOH (selenised methionol), non-selenised methionol was not observed. In previous studies on cauliflower and broccoli [29,30] incorporation of Se into the glucosinolates, and thus their aglycons, was very high; sometimes at equivalent concentrations to the non-selenised compounds. Herein, the percentage of Se incorporation into the radish GSLs and their aglycons was very much lower. If SeMet is not as readily taken up into the GSLs as Met is, then there may be an accumulated pool of SeMet available for biosynthesis of detectable amounts of selenised methionol.

We have previously reported 3MSeP-ITC and 4MSeB-ITC in broccoli [30], but this is the first report of 4MSeB3-ITC (experimental data in Section 2.2). Also 3MSePOH has not previously been reported in nature, although it has been produced synthetically [62]. 

As noted for 4MSeB3, selenised hydrolysis products were only detected in sprouts germinated from seeds of +Se plants, regardless of the presence of Se in the germination water (Table 3). This further confirmed that Se was not incorporated into the GSLs during the first 5 days of germination in this study.

Our investigation of GSL production during radish plant development confirmed the presence of all the aliphatic GSLs detected in the seeds and sprouts. However, this time 4MTB3 was the predominant GSL, followed by 4MSOB3 and 4MTB (Figure 6, A–D). The trend of increasing 4MTB3 concentration at the expense of 4MSOB3 during sprouting was also observed in the early stage of sprouting (Table 2), and has been previously described by Barillari et al. [52].

The significant decrease of the aliphatic GSLs overall during development (Figure 6) confirms previous findings that radish sprouts have significantly greater GSL content than mature tissues [63]. Selenium fertilisation did not appear to greatly alter the content of aliphatic GSLs in radish, as observed by Trolove et al. [12], though a significant decrease in 4MSOB3 contents at 21 and 29 days after germination (DAG) was observed in response to Se fertilisation (Figure 6B). Se treatment also tended to result in lower 4MTB3 contents compared with controls at all stages (Figure 6A) (*p* = 0.055), supporting a similar observation made by Schiavon et al. [3] who noted a significant decrease in 4MTB3 content in 45-day-old radish leaves following 1 week of hydroponic selenate treatment. 

The detection of the Se-containing GSL 4MSeB3 in plants fertilised with Se (Figure 6F) followed the production of 4MTB3 as the predominant aliphatic GSL found in radish [63] (Figure 6). It is expected that 4MSeB3 would be the most likely Se-GSL to be found in this species. 

The concentration of the predominant indole GSL, 4HOI3M, followed a similar pattern to the aliphatic GSLs, steadily decreasing with developmental stage (*p* < 0.001) and unaffected by Se treatment (Figure 7A). In contrast, the content of I3M significantly increased with developmental stage (*p* < 0.001, Figure 7B), and Se treatment caused a significant decrease in its concentration at 14 and 21 DAG (*p* < 0.001) compared to controls. Trolove et al. [12] also noted that Se addition significantly affected the content of indole GSLs in radish sprouts. In their research, Se addition decreased the content of 4HOI3M in 4-day-old sprouts and increased the content of 4MOI3M, unlike the decrease observed in this study, though this was not significant (Figure 7C). Trolove et al. [12] also found evidence of a difference between radish varieties in the effect of Se on I3M production, which may explain this difference between these studies. 

As expected, no indole seleno-GSLs were detected because these compounds are not derived from SeMet. Indeed, the great majority of Se-GSLs described thus far are aliphatic and have the Se atom in the position of the S donated by Met rather than in the sulfate group or on the bridge to glucose [29,30]. These compounds have presumably resulted from the incorporation of SeMet into the GSL backbone in place of Met. We have previously detected only a very small amount of one Se-GSL, seleno-(2-phenylethyl glucosinolate), in Se-fertilised broccoli where the Se atom is present in one of these other positions [30], indicating this is a rare event in Se-GSL production, and our current results in radish confirm this. This is disappointing as the synthetic production of selenosulforaphane (4-(methylsulfinyl)butyl isoselenocyanate), the theoretical breakdown product of selenoglucoraphanin (with a Se atom on the bridge to glucose), was found to be more effective against mouse HepG2 cancer cells [32] and human breast cancer cell lines [64], while being less toxic to non-cancer cells. However, this compound has not so far been found in nature. The effectiveness against cancer of the Se-GSLs found in this study and others is currently unknown.

When investigating the GSL hydrolysis products during radish plant development, in general it appeared that they declined, mirroring that of their parent GSL compounds, though their amounts were much lower than seen for the GSLs. This is probably due to incomplete hydrolysis, as during their extraction from the tissues some effort was made to suppress the myrosinase activity.

While the general trend for the GSLs and their hydrolysis products was to decrease on a FW basis during plant development, for all of the Se-GSL compounds detected here there was a significant increase in their production during development. From no detectable Se-GSLs in the –Se seed (Table 2) the concentration gradually increased in the radish plants signalling active production beginning with the formation of 4MSeB3-ITC from SeMet at 4 DAG (Figure 8G). 

*CYP79F1* and *CYP83A1* encode key enzymes involved in aliphatic GSL production [18]. *CYP79F1* encodes a cytochrome P450 enzyme, the first enzyme in the biosynthesis of the aliphatic core GSL structure, catalysing the conversion of chain-elongated methionine into its oxime. In Arabidopsis, the activity of this enzyme results in both short- and long-chain aliphatic GSLs and the gene is strongly expressed in cotyledons, rosette leaves, stems, and siliques [65]. The significant decrease in *CYP79F1* transcript abundance we observed during radish development (Figure 9A) was also noted by Gu et al. [53] following 7 days of radish growth, to a minimum at 21 days. The apparent decrease in *CYP79F1* transcript abundance on Se-fertilisation may be reflected in the significant decrease in 4MSOB3 content the Se-treated plants at 21 and 29 DAG (Figure 6B).

*CYP83A1* encodes another cytochrome P450 enzyme, the second enzyme in the biosynthesis of core GSL structure on the aliphatic biosynthetic pathway. The decline in transcript abundance of this gene on Se-fertilisation (Figure 9B) parallels that of *CYP79F1.* The observed decrease in the expression of two genes key to aliphatic GSL production upon Se fertilisation is interesting considering it is the aliphatic GSL forms that gain Se to form 4MSeB3 on exposure to Se (Figure 6F).

In Arabidopsis *CYP79B2* is the first enzyme in the production of the indole GSLs, catalysing the conversion of tryptophan to indole-3-acetaldoxime, the precursor of the indole glucosinolates. In Arabidopsis *CYP79B2* is induced by wounding and is expressed throughout the plant, with the highest expression in the roots [66]. *CYP83B1* encodes the enzyme following *CYP79B2* in the indole GSL biosynthetic pathway and in Arabidopsis is believed to be a key branch point enzyme between the primary auxin indole-3-acetic acid and indole glucosinolate biosynthesis [67]. The transcript abundance of both genes was observed to increase on Se treatment (Figure 9C,D). This observed increase in the expression of two genes encoding enzymes key to the biosynthesis of the indole GSLs on Se fertilisation is the reverse trend of what was observed for the genes encoding aliphatic GSL production enzymes (Figure 9A,B).

APS Kinase is a key branch point enzyme between the reductive S pathway and the secondary sulfate assimilation pathway, which uses 3′phosphoadenosine-5′-phosphosulfate (PAPS) as a S-donor for GSL biosynthesis [68]. The transcript abundance of this gene was significantly increased on Se fertilisation (Figure 9F). We have previously noted a similar increase in the transcript abundance of two *APS Kinase* homologues isolated from broccoli, *APSK1A* and *APSK2*, on increasing Se supply [69], implying this gene response to Se fertilisation may occur more broadly within the Brassicaceae family.

Of the eight genes investigated, the most significant changes to transcript abundance in response to Se treatment were observed for the four GSL biosynthetic genes (Figure 9A–D) and *APS kinase* (Figure 9F). It is interesting to consider why Se treatment would result in the induction of genes encoding the key branch point enzyme for GSL biosynthesis (APSK) and those involved in indole GSL biosynthesis, while simultaneously resulting in reduced transcript abundance of genes encoding enzymes involved in aliphatic GSL production. One possibility is that Se uptake by the plant, and particularly its incorporation into the GSLs, results in S being drawn into GSL biosynthesis by the up regulation of *APSK* gene expression, which was seen here in response to Se treatment across all developmental stages (Figure 9F). *APSK* transcription has been previously suggested as a means of controlling the amount of S-containing compounds following Se uptake in broccoli [69]. At the same time, the induction of gene transcripts favouring indole production, of which selenised forms have never been identified, at the expense of gene transcripts favouring aliphatic production, may form part of a general mechanism to protect radish, and possibly other Brassicaceae species, from the downstream effects of increased Se uptake, while still maintaining a full GSL complement. Indeed, we observed decreased 4MTB3 content (Figure 6A) and significantly decreased 4MSOB3 content (Figure 6B) at 21 and 29 DAG in radish plants fertilised with Se, although a corresponding increase in the indole GSLs was not observed (Figure 7).

Members of the Brassicaceae have evolved to be able to accumulate Se from the environment at concentrations of up to 0.1% (w/w) DW with minimal impact on their growth or development [70]. This is in part due to their ability to produce the Se-containing amino acid, MeSeCys, which effectively restricts Se from non-specifically entering the proteins via Se-Cys or Se-Met and therefore reduces Se-toxicity [5,71]. The described alteration in transcript abundance of GSL biosynthetic genes on Se-exposure may add to this general mechanism of protecting the plant from excess Se, while maintaining a GSL complement with minimal Se-GSLs present. In radish, 4MSeB3 production was particularly low as a percentage of total GSL concentration in the Se fertilised plants. At the developmental times this Se-GSL was detected (7, 21 and 29 DAG) it was 0.06, 0.13 and 1.4%, respectively, of the total GSL concentration. The increased percentage of this Se-GSL over successive developmental stages reflects both the increased concentration of 4MSeB3 and the general decrease of total GSL concentration over development (Figure 6). However, the proportion of Se-GSL content reported here is much lower than in many other brassica tissues we have investigated, suggesting that radish might not be a good candidate for the production of Se-GSLs. For example, the floret tissue of Se-fertilised broccoli had an equivalent concentration of Se-GSLs to their non-selenised forms [31]. However, the bioactivity of Se-GSLs such as 4MSeB3 still needs to be determined especially in the context of the reported bioactivity of the non-selenised hydrolysis product, 4MTB3 [72,73,74], and the bioactivity of synthetically produced Se-GSL breakdown products, such as Se-containing sulforaphane, which are reported to be extremely effective in animal and cell model systems [32,64]. 

## 4. Materials and Methods

### 4.1. Plant Material and Growth Conditions

#### 4.1.1. Production of Se-enriched Seeds and Sprouts

Fifteen radish plants (*Raphanus sativus* L. cv. ‘Rambo’, Kings Seeds, Katikati, New Zealand) were grown in sand. The plants were fertilised with Nitrophoska Blue TE + Mg and B (Ravensdown Fertiliser Co-operative Ltd., New Zealand) following germination and at the beginning of flowering. From mid-flowering until the end of seed ripening Se was applied to the soil as 20 µM sodium selenate every 2 days. A total of 180 L of Se solution was applied. Siliques were harvested when dry and the seeds removed.

#### 4.1.2. Production of 5-day-old Sprouts from Control and Se-enriched Radish Seeds

Sprouts were produced according to the method recommended by Trolove et al. [12]. Briefly, 3.5 g of control seed (-Se seed) or Se-enriched seed (+Se seed) was weighed and placed into 500 mL plastic containers equipped with a 3 mm hole for gas exchange. The +Se seed contained 174 µg Se g^−1^ DW (Table 1). Fifty mL of 2 g L^−1^ sodium hypochlorite was added with stirring for 20 min to surface sterilise the seed, before being poured off and 50 mL sterile water (−Se water), or 50 mL of water containing 609 µg Se (the same amount of Se as in the 3.5 g of seed in the +Se seed treatment) as sodium selenate (+Se water) added. The containers were rolled end-over-end at six rpm for 4 days at 24 °C. The sprouts were further washed with 75 µg mL^−1^ chlorine as sodium hypochlorite. There were three replicates of each treatment. Material from each replicate was blotted dry and separated into three accurately weighed amounts for the separate analysis of total Se content, GSL content and the production of GSL hydrolysis products. The material was frozen in liquid nitrogen and stored at −80 °C until analysis.

#### 4.1.3. Production of Plant Material for Radish Developmental Series

Radish plants were grown under hydroponic conditions in a greenhouse with a mean day/ night temperature of 22/17 °C. Radish seed (10 g) was sprinkled over a rockwool pad soaked in half strength Hoagland’s solution [75] which was aerated and circulated continuously through a 40 L reservoir tank. The nutrient solution was changed every 7 days. Six independent systems were run, one for each of three control replicates and three Se-enriched treatments. Selenium was supplied as sodium selenate, at a concentration of 40 µM for the duration of the experiment. Plant material was harvested from each system at 4, 7, 14, 21 and 29 days after application of the seed to the saturated rockwool. As the seed began to germinate within 24 h of sowing, these times are referred to as 4, 7, 14, 21 and 29 days after germination (DAG) and correspond to the developmental stages; sprouts (4 DAG), sprouts with fully expanded cotyledons (7 DAG), microgreens (first two true leaves present, 14 DAG), salad greens (first two true leaves fully expanded, 21 DAG) and mature greens (several pairs of fully expanded true leaves, 29 DAG). Sprout and shoot material was harvested at each developmental stage, frozen in liquid nitrogen and stored at −80 °C until analysis.

### 4.2. Selenium Analysis

Seed and sprouts were freeze dried, ground and digested with tetramethylammonium hydroxide at 90 °C for 1 h, and total Se determined by ICP-MS at Hill Laboratories Limited (Hamilton, New Zealand) based upon Anderson [76]. 

### 4.3. Preparation of Plant Material for Liquid Chromatography-Mass Spectrometry and Gas Chromatography-Mass Spectrometry Analysis

Plant material was weighed, frozen in liquid nitrogen and ground to a fine powder in a mortar and pestle before being extracted according to Matich et al., [30]. There was only sufficient +Se and –Se seed available for a single sample of 2–3 g FW. For the sprout material, three replicates of frozen plant material for each of the four Se treatments (-Se seed/-Se water, -Se seed/+Se water, +Se seed/-Se water, +Se seed/+Se water) were extracted. 

### 4.4. Analysis of Glucosinolates in Radish Tissues by Liquid Chromatography-Mass Spectrometry 

Separations were performed on an Ultimate^®^ 3000 Rapid Separation LC (Dionex, Germering, Germany) with detection in a micrOTOF-Q II mass spectrometer (Bruker Daltonics, Bremen, Germany) fitted with an electrospray source operating in negative mode. Sample injections (1 µL) were made onto two 50 mm × 2.1 mm i.d., 1.8 µm Zorbax™ SB-C18 (Agilent) analytical columns (in series) maintained at 60 °C and with a flow of 350 µL min^−1^. Solvent A was MeCN, and Solvent B was HCO_2_H:H_2_O (0.2:99.8). The solvent ramp was 10:90 (Solvent A:Solvent B) from injection to 5 min, a linear gradient to 40:60 (5–15 min), to 100:0 (15–18 min), and held at 100:0 (18–25 min). The micrOTOF-Q II source parameters were: temp. 200 °C; drying N_2_ flow 8 L min^−1^; nebuliser N_2_ 4 bar (400 kPa); endplate offset −500 V; capillary voltage +3500 V; mass range 100–1500 Da at 2 scans s^−1^. For MS/MS nitrogen gas was used in the collision cell, and the ionisation energy was 26 eV. Post-acquisition internal mass calibration used sodium formate clusters with the sodium formate delivered by a syringe pump at the start of each analysis. Mass spectral data were processed by Compass DataAnalysis (Bruker Daltonics). The external standard used for quantification was 4MTB; Cfm Oskar Tropitzsch, Marktredwitz, Germany). [M-H]^−^ ions were used for quantification. The external standard and the plant extracts were spiked with an internal standard, epicatechin (Sigma-Aldrich, Auckland, New Zealand) to enable correction for sample volumes and for possible changes in LC-MS system responses. Identification of the methylthio- and methylseleno-glucosinolates by high-resolution-LC-MS/MS has been described previously [29]. 

### 4.5. Analysis of Glucosinolate Hydrolysis Products in Radish Tissues by GC-MS

Frozen powdered tissue, prepared as described in Section 4.3, was extracted into cold diethyl ether as described previously [30]. Separations were performed on an Agilent 6890N GC coupled to a Waters GCT time of flight (ToF) mass spectrometer with EI energy of 70 eV and a scan time of 0.4 s. Splitless injections of 1 µL over 30 s were made at 220 °C onto a 20 m × 0.18 mm i.d. × 0.18 µm film thickness, DB-Wax^®^ (Agilent) capillary column with a He flow of 0.9 mL min^−1^. The oven temperature programme was 1 min at 35 °C, 3 °C min^−1^ to 100 °C, 7 °C min^−1^ to 240 °C, and held for 5 min. Compounds were quantified against the synthetic external standard 5-(methylseleno)pentanenitrile (CH_3_SeC_4_H_8_CN). To keep concentrations of compounds in the solvent extracts in the linear range of the MS detector, samples were injected neat, and at up to 500-fold dilutions. Compound identifications and synthesis of the 5-(methylseleno)pentanenitrile external standard have been described previously [29].

### 4.6. RNA Extraction and Quantitative Real Time-Polymerase Chain Reaction (qRT-PCR)

Total RNA was isolated from four stages of the radish developmental series (1, 4, 14 and 21 DAG) and qRT-PCR was performed as described in Esfandiari et al. [77]. Transcriptional abundance was normalized to *ACTIN7* using the ΔΔCt method [78]. Radish orthologs of Arabidopsis glucosinolate-related genes were identified at the RadishBase website using BLAST (http://bioinfo.bti.cornell.edu/cgi-bin/radish/tool/blast.cgi). Gene transcript abundance was quantified by qRT-PCR using primers designed with PrimerQuest software (www.idtdna.com). Gene targets together with their RadishBase ID, closest GenBank ID and qRT-PCR primer sequences are listed in Table S1.

### 4.7. Statistical Analyses

Chemical concentrations and gene expression were analysed using analysis of variance, with Time after Germination and Se treatment (plus interaction) as the two factors. Concentrations were log transformed before analysis to make the variability of the data more even. The gene expression data did not require this. Analysis was completed with Genstat (version 14, VSNi Ltd., Hemel Hempstead, UK, 2011).

## Figures and Tables

**Table 1:** Total mean selenium (Se) content in *Raphanus sativus* cv. ‘Rambo’ seed sourced from control and Se-fertilised plants, and in 5-day-old sprouts produced from the same seed following germination in the presence or absence of Se.

Tissue Type	Source and Treatment	Total Se Content (µg g^−1^ DW ^a^)
Seed	− Se seed ^b^	0.1 ± ^c^ 0.02
Seed	+ Se seed ^d^	174 ± 1.0
Sprout ^e^	− Se seed / − Se water	0.21 ± 0.03
Sprout	− Se seed / + Se water	229.8 ± 20.9
Sprout	+ Se seed / − Se water	187.1 ± 2.9
Sprout	+ Se seed / + Se water	335.0 ± 32.0

^a^ DW, dry weight. ^b^ data are the mean of three replicates, each comprising 3 g seed. ^c^ ± represents the standard error of the mean. ^d^ data are the mean of two replicates, each comprising 1.3 g seed. ^e^ sprout data are the mean of three replicate samples, each sample comprising 2–5 g sprouts.

**Table 2:** Mean glucosinolate content in *Raphanus sativus* cv. ‘Rambo’ seed sourced from control and selenium (Se)-fertilised plants, and in 5-day-old sprouts produced from the same seed, following germination in the presence or absence of Se. Glucosinolates are listed in order of most to least abundant, with the Se-containing glucosinolate reported last.

(µg g^−1^ FW ^a^)	Seed Source ^b^		Sprout Source ^c^			
Glucosinolate	− Se seed	+ Se seed	− Se seed − Se water	− Se seed + Se water	+ Se seed − Se water	+ Se seed + Se water
4MSOB3	1097.0	1696.0	676 ± ^d^ 45	708 ± 43	713 ± 55	721 ± 139
4MTB3 b	81.0	177.0	308 ± 50	248 ± 23	269 ± 37	231 ± 57
4MTB	16.3	26.6	14.3 ± 1.5	13.2 ± 1.4	16.0 ± 2.0	15.0 ± 3.6
4MTB3 a	4.5	9.7	3.7 ± 0.4	4.17 ± 0.59	7.0 ± 1.1	6.64 ± 0.75
possible 4MSeB3	nd ^e^	7.8	nd	nd	2.9 ± 0.2	2.7 ± 0.7
Total glucosinolates	1198.8	1917.1	1002 ± 96.9	973.4 ± 67.9	1007.9 ± 5.3	976.3 ± 2010

^a^ FW, fresh weight, ^b^ seed data comprises a single replicate of 2–3 g seed, ^c^ sprout data comprises three replicate samples, each sample comprising 7–12 g sprouts, ^d^ ± represents the standard error of the mean, ^e^ nd, not detected. 4MSOB3: 4-(methylsulfinyl)but-3-enyl, 4MTB3 b: 4-(methylthio)but-3-enyl isomer b, 4MTB: 4-(methylthio)butyl, 4MTB3 a: 4-(methylthio)but-3-enyl isomer a, 4MSeB3: 4-(methylseleno)but-3-enyl.

**Table 3:** Mean nitrile and isothiocyanate hydrolysis product (modified aglycons) content in *Raphanus sativus* cv. ‘Rambo’ seed sourced from control and selenium (Se)-fertilised plants, and in 5-day-old sprouts produced from the same seed, following germination in the presence or absence of Se. Compounds are listed in order of most to least abundant, with the Se-containing compounds reported last.

Compound (µg g^−1^ FW ^a^)	Seed Source ^b^		Sprout Source ^c^			
	− Se seed	+ Se seed	− Se seed − Se water	− Se seed + Se water	+ Se seed − Se water	+ Se seed + Se water
4MTB3-ITC b	0.07	2.6	283 ± ^d^ 155	151 ± 49	208 ± 7	208 ± 32
4MTB-ITC	0.04	0.32	7.72 ± 3.25	2.47 ± 0.53	6.92 ± 0.43	5.5 ± 2.58
4MTB3-ITC a	0.01	0.1	1.86 ± 0.35	2.09 ± 0.34	1.75 ± 0.24	1.26 ± 0.64
3MTP-ITC	nd ^e^	0.07	1.31 ± 0.1	1.56 ± 0.63	0.9 ± 0.08	0.67 ± 0.24
5MTP-ITC	nd	nd	0.11 ± 0.02	0.12 ± 0.04	0.13 ± 0.01	0.11 ± 0.01
possible 4MSeB3-ITC b	nd	0.04	nd	nd	1.08 ± 0.08	1.05 ± 0.11
3MSeP-ITC	nd	0.013	nd	nd	0.14 ± 0.01	0.13 ± 0.01
possible 4MSeB3-ITC a	nd	nd	nd	nd	0.05 ± 0.001	0.04 ± 0.006
3MSePOH	nd	nd	nd	nd	0.03 ± 0.003	0.09 ± 0.01
4MSeB-ITC	nd	nd	nd	nd	0.01 ± 0.001	0.01 ± 0.001
Total aglycons	0.12	3.14	294 ± 159	157 ± 50	219 ± 7.9	216 ± 36

^a^ FW, fresh weight, ^b^ seed data comprises a single replicate of 2–3 g seed, ^c^ sprout data comprises three replicate samples, each sample comprising 7–12 g sprouts, ^d^ ± represents the standard error of the mean, ^e^ nd, not detected. 4MTB3-ITC b: 4-(methylthio)but-3-enyl isothiocyanate isomer b, 4MTB-ITC: 4-(methylthio)butyl isothiocyanate, 4MTB3-ITC a: 4-(methylthio)but-3-enyl isothiocyanate isomer a, 3MTP-ITC: 3-(methylthio)propyl isothiocyanate, 5MTP-ITC: 5-(methylthio)pentyl isothiocyanate, 4MSeB3-ITC b: 4-(methylseleno)but-3-enyl isothiocyanate isomer b, 3MSeP-ITC: 3-(methylseleno)propyl isothiocyanate, 4MSeB3-ITC a: 4-(methylseleno)but-3-enyl isothiocyanate isomer a, 4MSeB-ITC: 4-(methylseleno)butyl isothiocyanate, 3MSePOH: 3-(methylseleno)propanol.

## Supplementary Materials

The following are available online at https://www.mdpi.com/2223-7747/8/10/427/s1, **Table S1.** Quantitative Real Time PCR (qRT-PCR) primers used for quantifying glucosinolate biosynthetic and hydrolysis-related gene transcript abundance in *Raphanus sativus*. **Figure S1.** Selenium isotope clusters for the tentatively identified 4MSeB3 pseudomolecular ion and its major selenium-containing daughter ion. **Figure S2.** EI-MS fragmentation patterns of the two isomers of 4MTB3-ITC. **Figure S3.** EI-MS fragmentation patterns of the two isomers of the proposed compound 4MSeB3-ITC.

## Abbreviations

3′phosphoadenosine-5′-phosphosulfate (PAPS), adenosine 5′-phosphosulfate (APS), APS kinase (APSK), APS reductase (APR), cysteine (Cys), days after germination (DAG), dry weight (DW), fresh weight (FW), Gas Chromatography-Mass Spectrometry (GC-MS), tandem mass spectrometry (MS/MS), electron ionization (EI), nuclear magnetic resonance (NMR), glucosinolate (GSL), indol-3-ylmethyl glucosinolate (I3M), 4-hydroxyindol-3-ylmethyl glucosinolate (4HOI3M), 4-methoxyindol-3-ylmethyl glucosinolate (4MOI3M), 4-(methylthiobutyl) glucosinolate (4MTB), 3-(methylsulfinylpropyl) glucosinolate (3MSOP), 3-(methylseleno)propyl (3MSeP), 4-(methylseleno)but-3-enyl glucosinolate (4MSeB3), 4-(methylsulfinyl)but-3-enyl glucosinolate (4MSOB3), 4-(methylseleninyl)butyl glucosinolate (4MSeOB), 4-(methylthio)but-3-enyl glucosinolate (4MTB3), isothiocyanate (ITC), Liquid Chromatography-Mass Spectrometry (LC-MS), methionine (Met), methylselenocysteine (MeSeCys), nitrile specifier protein (NSP), quantitative Real Time-Polymerase Chain Reaction (qRT-PCR), selenocysteine (SeCys), selenoglucosinolate (Se-GSL), selenomethionine (SeMet)

## Appendix A

**Table A1:** **Structures of the side chains for glucosinolates reported in *Raphanus sativus* tissues.** The glucosinolate group in the glucosinolate is represented by “X”. Descriptive chemical names, their abbreviations, and commonly used trivial names, and their abbreviations, are included.

Glucosinolate Side Chain	Chemical Name (Abbreviation)	Trivial Name (Abbreviation)
	3-(methylthio)propyl-GSL (3MTP)	glucoibervirin (GIB) glucoiberverin
	3-(methylsulfinyl)propyl-GSL (3MSOP)	glucoiberin (GI)
	but-3-enyl-GSL (B3)	gluconapin (GNP)
	2-hydroxybut-3-enyl-GSL (2HOB3)	progoitrin (PRO)
	4-(methylthio)but-3-enyl-GSL (4MTB3)	dehydroglucoerucin glucoraphasatin (GRH)
	4-(methylsulfinyl)-GSL but-3-enyl- (4MSOB3)	glucoraphenin (GRE)
	4-(methylthio)butyl-GSL (4MTB)	glucoerucin (GER)
	4-(methylsulfinyl)butyl-GSL (4MSOB)	glucoraphanin (GRA)
	R = H indol-3-ylmethyl-GSL (I3M)	glucobrassicin (GBS)
	R = OH 4-hydroxyindol-3-ylmethyl-GSL (4HOI3M)	4-hydroxyglucobrassicin (4HGBS)
	R = OCH_3_ 4-methoxyindol-3-ylmethyl-GSL (4MOI3M)	4-methoxyglucobrassicin (4MGBS)
