# Supplementary material for: Selenium Application During Radish (Raphanus sativus) Plant Development Alters Glucosinolate Metabolic Gene Expression and Results in the Production of 4-(methylseleno)but-3-enyl glucosinolate
Source: Plants (Basel). 2019 Oct 18;8(10):427. doi: 10.3390/plants8100427 (PMC6843385; doi:10.3390/plants8100427)
Supplement: Supplementary file 1 [file plants-08-00427-s001.pdf]

Table S1. Quantitative Real Time PCR (qRT-PCR) primers used for quantifying glucosinolate biosynthetic and hydrolysis-related gene transcript abundance in *Raphanus sativus*.

Gene target	RadishBase ID[a]	GeneBank ID	Description	Pathway	Primer(5′-3′)
CYP79B1	UN28455	XM_018591728.1	Cytochrome P450 Tryptophan N-monooxygenase	Indolic (synthesis)	F AAGCAGCTAAACACCGAGATAG<br>R GTCATAGGTCTTGAGGCGAATAG
CYP83B1	UN51198	XM_018603411.1	cytochrome P450	Indolic (synthesis)	F GACATCCCAAATCTCCCTTATCT<br>R GCGTCTGCTATGGTTTCTCTAT
CYP79F1	UN19600	XM_018584676.1	cytochrome P450 dihomomethionine-N-hydroxylase	Aliphatic (synthesis)	F TACATTACCACCCTCTCCTCTT<br>R GAGTTTCTCTCCGGCTGATTAC
CYP83A1	UN22968	XM_018629165.1	cytochrome P450	Aliphatic (synthesis)	F GGAAACCAAACCAAAGAGCATAG<br>R CGGAGAAAGTGAGCAAGTGATA
MYROSINASE4	UN18772	XM_018634237.1	myrosinase	hydrolysis	F CCTTTGGATGCGAGAGAGTAAG<br>R GGCTTCTTAAGCCCACGTATAG
NITRILE-SPECIFIER	UN26475	XM_018584970.1	nitrile specifier	hydrolysis	F CCAGCCCTTTCTTACCATCAA<br>R GAAGTGGGAGAGGTTGGATAAG
APS kinase	UN65099	XM_018630545.1	5′-adenylylsulfate kinase	synthesis	F TCGGAAACTCGACGAACATAAA<br>R ACTAAGACCAGTGACCCAAATC
MYB28	UN76555	XM_018596832.1	Transcription factor Myb-related protein 28	Aliphatic (synthesis)	F TCGGTCATAGCGAGACATTTAC<br>R TTGTGAGTCACGGGATCAATAC
ACTIN7	UN84481	XM_018620829.1	Actin2/7	reference	F CCTGGATAGCAACATACAT<br>R GCATCACACTTTCTACAAC

[a] http://bioinfo.bti.cornell.edu/cgi-bin/radish/EST/

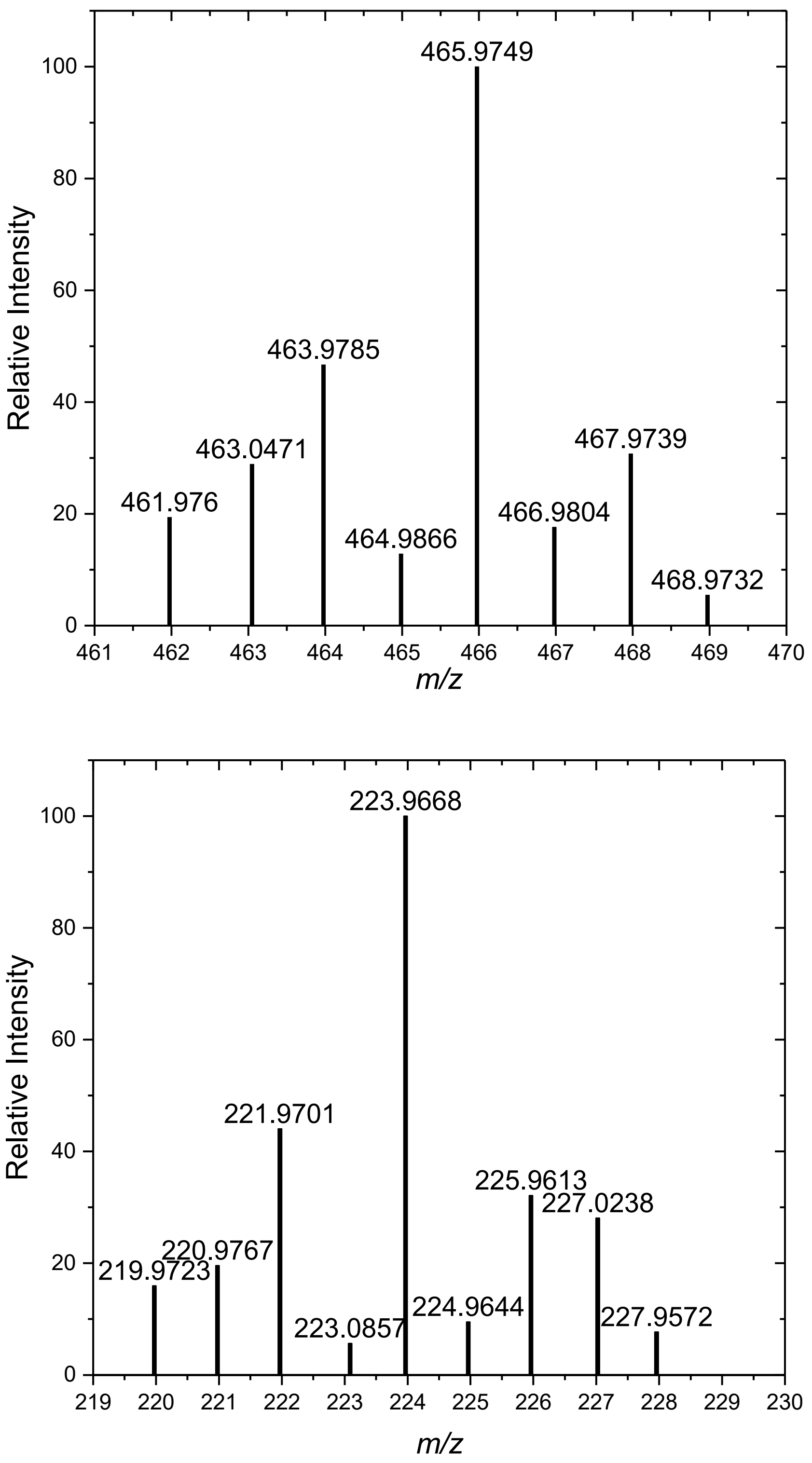

Figure 1. Selenium isotope clusters for the tentatively identified 4MSeB3 pseudomolecular ion and its major selenium-containing daughter ion.

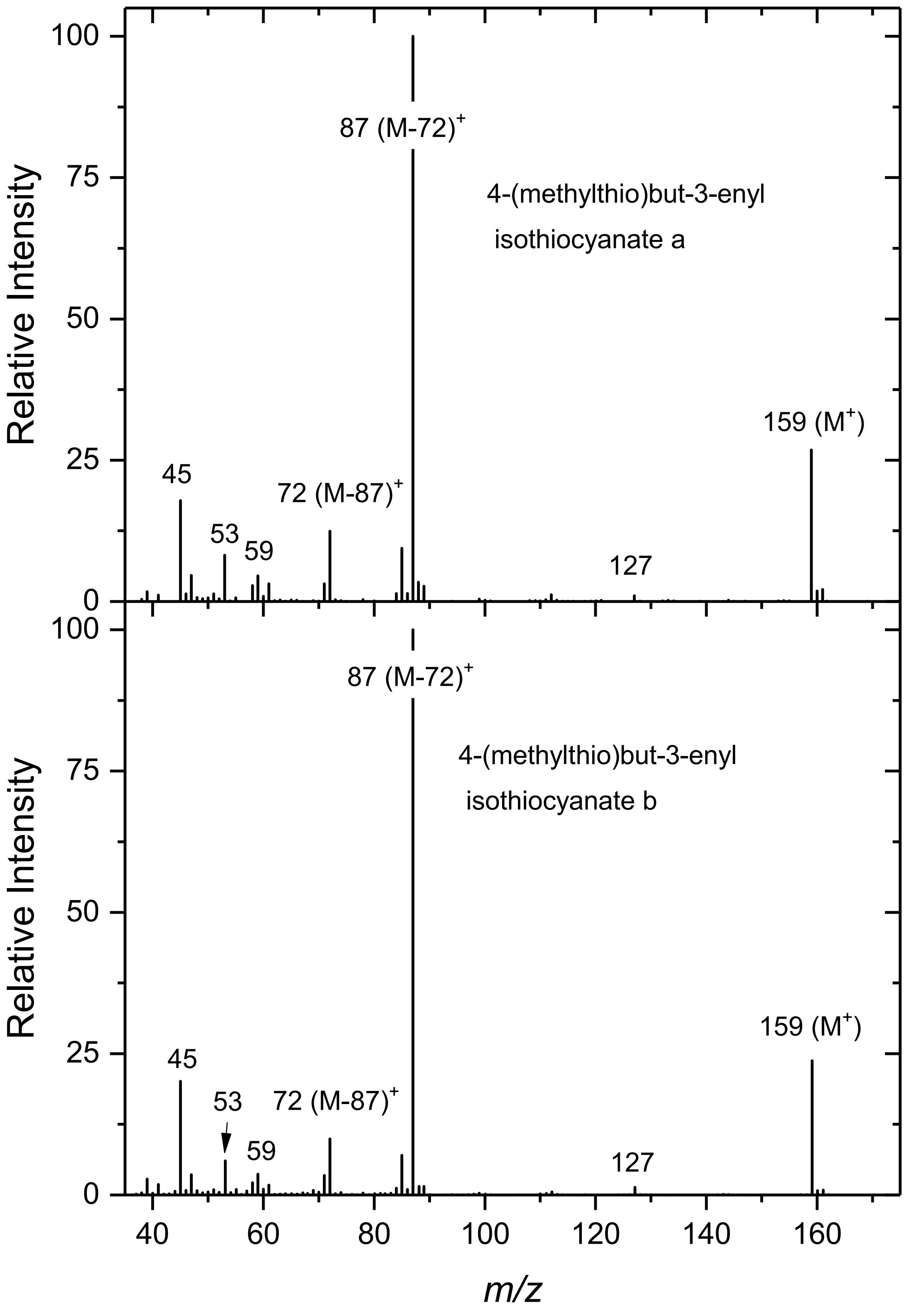

Figure 2. EI-MS fragmentation patterns of the two isomers of 4MTB3-ITC.

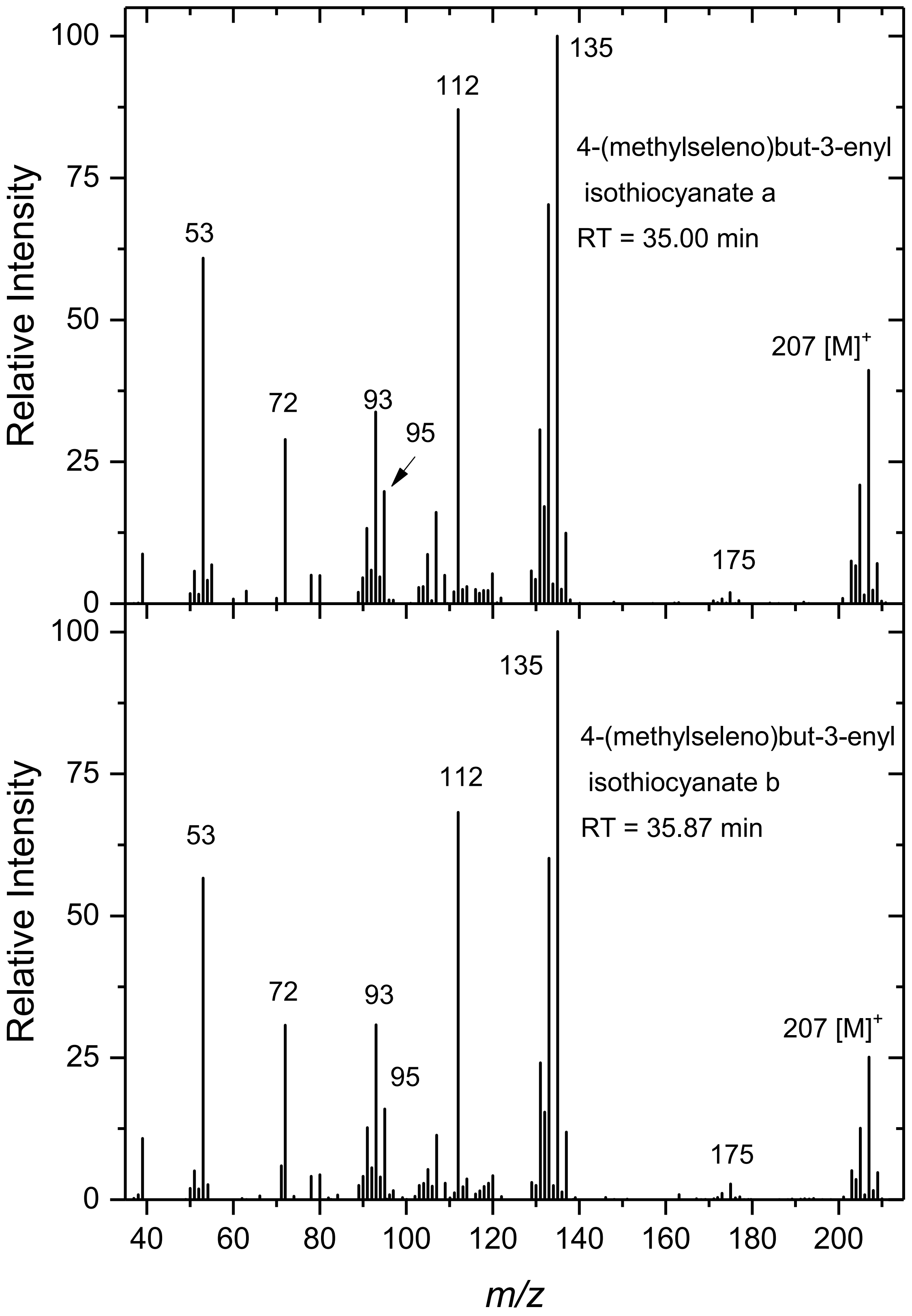

Figure 3. EI-MS fragmentation patterns of the two isomers of the proposed compound 4MSeB3-ITC.
